# Fluoroquinolone-associated suspected tendonitis and tendon rupture: A pharmacovigilance analysis from 2016 to 2021 based on the FAERS database

**DOI:** 10.3389/fphar.2022.990241

**Published:** 2022-09-06

**Authors:** Yamin Shu, Qilin Zhang, Xucheng He, Yanxin Liu, Pan Wu, Li Chen

**Affiliations:** ^1^ Department of Pharmacy, Tongji Hospital, Tongji Medical College, Huazhong University of Science and Technology, Wuhan, China; ^2^ Department of Pharmacy, Union Hospital, Tongji Medical College, Huazhong University of Science and Technology, Wuhan, China; ^3^ Department of Pharmacy, Pengzhou Second People’s Hospital, Pengzhou, China; ^4^ Department of Pharmacy, Pengzhou People’s Hospital, Pengzhou, China; ^5^ Department of Pharmacy, Chengfei Hospital, Chengdu, China; ^6^ Department of Pharmacy and Evidence-Based Pharmacy Center, West China Second University Hospital, Sichuan University, Chengdu, China

**Keywords:** data mining, FAERS, fluoroquinolones, pharmacovigilance, tendonitis, tendon rupture

## Abstract

**Objective:** The objective of this study was to scientifically and systematically explore the association between fluoroquinolones (ciprofloxacin, levofloxacin, and moxifloxacin) and tendonitis and tendon rupture through the Food and Drug Administration Adverse Event Reporting System (FAERS) database.

**Methods:** Disproportionality analysis was used to quantify the signals of fluoroquinolone-associated suspected tendonitis and tendon rupture based on the FAERS data from January 2016 to March 2021. Clinical characteristics, the onset time, oral and intravenous administrations, and the serious outcomes of fluoroquinolone-associated tendonitis and tendon rupture were further analyzed.

**Results:** Out of 35,667 fluoroquinolone-associated adverse events recorded in the FAERS database during the study period, 1,771 tendonitis and 1,018 tendon ruptures induced by fluoroquinolones as the suspected drug were analyzed, with a median age of 49.88–63.87 years. All three fluoroquinolones detected positive signals of tendonitis and tendon rupture in the four methods. Ciprofloxacin had the strongest statistical association with tendonitis with the highest positive signal values (ROR 98.50, PRR 93.25, IC 6.15, and EBGM 76.80), while levofloxacin showed the strongest statistical association with tendon rupture (ROR 76.38, PRR 73.75, IC 5.84, and EBGM 63.89). Compared with ciprofloxacin and levofloxacin, moxifloxacin was relatively weakly associated with tendonitis and tendon rupture. Oral fluoroquinolone-induced tendonitis and tendon rupture had a stronger signal strength than intravenous administration. The majority of fluroquinolone-related suspected tendonitis and tendon rupture tended to occur within a few days or one month. As for the disability rate of tendonitis, ciprofloxacin counted the highest (*n* = 461, 50.94%), with moxifloxacin the lowest (*n* = 20, 29.41%).

**Conclusion:** Fluoroquinolone-induced tendonitis and tendon rupture tended to occur early and might result in serious outcomes. Our study provided valuable references for early identification of the risk of fluoroquinolone-induced tendonitis and tendon rupture.

## Introduction

Fluoroquinolones are a kind of synthetic broad-spectrum antibacterial agents, which are widely used in the treatment of gastrointestinal, respiratory, genitourinary, and ophthalmic infections due to their advantages of high oral bioavailability, excellent tissue penetration, and long half-life (Redgrave et al., 2014). Fluoroquinolones exert distinctive antibacterial effects by inhibiting DNA gyrase and topoisomerase IV, thus inhibiting synthesis of bacterial DNA directly (Hawkey, 2003; Owens and Ambrose, 2005). The third- and fourth-generation fluoroquinolones commonly used in clinical practice, such as ciprofloxacin, ofloxacin, levofloxacin, moxifloxacin, and gatifloxacin, have significantly enhanced antibacterial activity compared with previous generations, especially against Gram-positive bacteria and anaerobes (Millanao et al., 2021).

With the widespread use of fluoroquinolones, the adverse drug reaction (ADR) reports are increasing gradually, which has attracted the attention of physicians and drug administrations. The most commonly reported adverse events (AEs) related to fluoroquinolones include gastrointestinal discomfort (nausea and diarrhea), psychiatric (anxiety and depression) and nervous system disturbances (headache and dizziness), and cardiovascular symptoms (Goa et al., 1997; Saravolatz and Leggett, 2003; Meng et al., 2019; Morales D. et al., 2019). Hepatotoxicity, ototoxicity, hypoglycemia and hyperglycemia, anaphylaxis, sensitivity to light, tendonitis, and tendon rupture could also be reported with fluoroquinolones (Paterson et al., 2012; Chou et al., 2013; Arabyat et al., 2015; Kounis et al., 2017). The US Food and Drug Administration (FDA) approved the revised specifications for quinolones in 2016. As amended in the black box warnings and the warnings and precautions sections in the drug label, common serious ADRs associated with quinolones include tendonitis, tendon rupture, arthralgia, myalgia, peripheral neuropathy, and central nervous system effects. Among them, tendonitis and tendon rupture can lead to long-term sequelae, including chronic pain and mobility restrictions, and may require surgery (Bidell and Lodise, 2016).

Although there have been systematic literature reviews, cohorts, and case–control studies on fluoroquinolone-related tendon rupture, the data are old and the number of reports is limited (Khaliq and Zhanel, 2003; Corrao et al., 2006; Sode et al., 2007). A case–control study based on a database of British population showed that fluoroquinolone exposure was associated with an increased risk of any tendon rupture, which was consistent with a cohort study in Taiwan (Morales D. R. et al., 2019; Chang et al., 2022). However, Baik et al. (2020) reported the contrary results that fluoroquinolones as a class were not associated with the increased risk of tendon ruptures. The different results might be due to differences in study design and population. Furthermore, the relationship between oral and intravenous fluoroquinolone-induced tendonitis and tendon rupture risk is unclear. In addition, the seriousness and onset of various fluoroquinolone-related tendonitis and tendon rupture also require further research, and there are no latest systematic reviews on these studies. To further explore this serious adverse event, a retrospective study of an international large-sample pharmacovigilance database was conducted to analyze reports of tendonitis and tendon rupture with FDA-approved and marketed fluoroquinolones (ciprofloxacin, levofloxacin, and moxifloxacin), which were widely used in clinical practice from the FDA Adverse Event Reporting System (FAERS) database.

Recently, drug safety assessment through data mining of a large adverse event spontaneous reporting system database has become an important means of pharmacovigilance research. The FAERS is a public, accessible, and free database in the United States that contains tens of millions of AE reports voluntarily submitted by health professionals, consumers, manufacturers, and others, which is designed to support the FDA’s safety monitoring for post-marketing drug and biological products (Cirmi et al., 2020; Hu et al., 2020). This study aimed to quantitatively measure AE signal intensity by disproportionality analysis and to assess the risk of tendonitis and tendon rupture caused by different fluoroquinolones.

## Materials and methods

### Data sources

**FIGURE 1:**
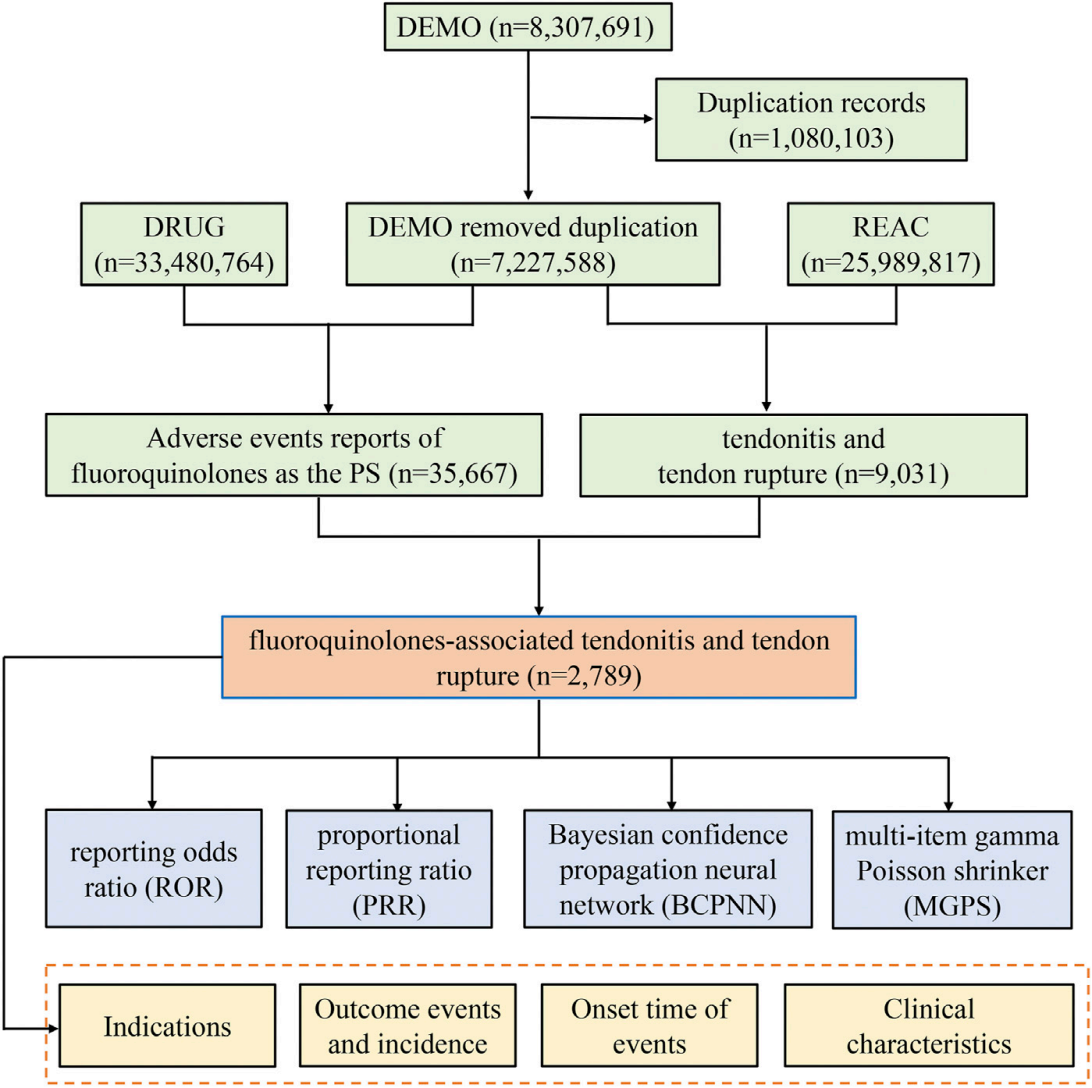
The process of selecting fluoroquinolone-associated tendonitis and tendon rupture from Food and Drug Administration adverse event reporting system (FAERS) database.

The FAERS database is updated every quarter, and users can download data in the XML or ASCII format for free from the FDA website. The FAERS database of every quarterly file package includes the following seven data files: patient demographic and administrative information (DEMO), drug information (DRUG), coded for the adverse events (REAC), patient outcomes (OUTC), report sources (RPSR), therapy start dates and end dates for reported drugs (THER), and indications for drug administration (INDI), and deleted cases. The files record all relevant information about AEs in detail, associated with PRIMARYID, CASEID, and drug_seq. In our study, the reports submitted from January 2016 to March 2021 in the FAERS database were extracted. The deduplication process was performed before statistical analysis according to FDA recommendations, by selecting the latest FDA_DT when CASEIDs were the same, and choosing the higher PRIMARYID when the CASEID and FDA_DT were the same, resulting in a reduction in the number to 7,227,588 (Figure 1).

### Procedures

The AEs in REAC files were encoded by the preferred terms (PTs) in the Medical Dictionary for Regulatory Activities 24.0 (MedDRA). In order to improve accuracy, we limited our analysis reports to those in which the role_cod of drug was “PS” (primary suspected) in the DRUG files. Tendonitis and tendon rupture were identified by the PT code numbers 10043255 and 10043248. The generic and trade names of three fluoroquinolones were selected through drugs@FDA. Clinical characteristics (gender, age, reporting area, reporter, concomitant drug, and indication, etc.) of reports with fluoroquinolone-associated tendonitis and tendon rupture were collected. In addition, the time-to-onset of tendonitis and tendon rupture and the proportion of serious outcomes caused by different fluoroquinolones were calculated. The onset time is defined as the interval between EVENT_DT (date of AE occurrence) and START_DT (start date for fluoroquinolone use). Reports with input errors (EVENT_DT earlier than START_DT), inaccurate date entries, and missing specific data were excluded (Shu et al., 2022). Severe outcomes mainly included life-threatening events or those causing hospitalization, disability, or death. The proportion was calculated by dividing the number of serious outcomes by the total number of reports.

### Data mining

The disproportionality analysis, a fundamental tool of analytic methods in pharmacovigilance, was employed to detect safety signals, by using the reporting odds ratio (ROR), the proportional reporting ratio (PRR), the information component (IC), and the empirical Bayes geometric mean (EBGM) (van Puijenbroek et al., 2002; Shu et al., 2022). When a target drug is more likely to induce a target AE than all other drugs, it will typically get a higher score due to a higher disproportionality. The equations and criteria for the four algorithms are shown in Supplementary Table S1. One of the four algorithms that meet the criteria should be considered a positive signal of tendonitis or tendon rupture. Moreover, the relationship between oral or intravenous administration of fluoroquinolones (ciprofloxacin, levofloxacin, and moxifloxacin) and tendonitis or tendon rupture was studied in our work, and the rank order of the association was determined. In addition, we also performed subgroup analysis to check the strength of signal in male/female and different age (18≤and≤65 years, >65 years) groups. All data processing and statistical analyses were performed using MYSQL 8.0, Navicat Premium 15, Microsoft Excel 2019, and GraphPad Prism 8 (GraphPad Software, CA, United States).

## Results

### Descriptive analysis

A total of 35,667 fluoroquinolone-associated AEs were recorded in the FAERS database from January 2016 to March 2021, among which 1,771 were for tendonitis and 1,018 were for tendon rupture. Different fluoroquinolones have variable distribution of reported clinical characteristics, which are described in Table 1. The most commonly reported tendinitis was treated with ciprofloxacin (*n* = 905, 51.10%), followed by levofloxacin (*n* = 798, 45.06%) and moxifloxacin (*n* = 68, 3.84%). Specifically, 536 (52.65%) tendon rupture pertained to levofloxacin, 449 (44.11%) to ciprofloxacin, and 33 (3.24%) to moxifloxacin. Compared with ciprofloxacin and levofloxacin, moxifloxacin reported a very small reporting frequency of tendonitis and tendon rupture. Tendonitis and tendon rupture induced by the three fluoroquinolones were generally reported more frequently in females than in males. The mean age of patients ranged from 49.88 to 63.78 years. The most reported therapeutic indication for all fluoroquinolones was infections. Tendonitis and tendon rupture are rarely fatal or life-threatening, and the majority of patients showed severe outcomes such as disability and prolonged hospitalization as a result of fluoroquinolone-induced tendonitis and tendon rupture. As for the disability rate of tendonitis, ciprofloxacin counted the highest (*n* = 461, 50.94%), with moxifloxacin the lowest (*n* = 20, 29.41%). Among fluoroquinolone-induced tendon ruptures leading to hospitalization or prolonged hospitalization, moxifloxacin was the most frequently reported (*n* = 14, 42.42%). The vast majority of reports came from Europe and North America. The top five concomitant drugs for fluoroquinolone-associated tendonitis and tendon rupture are listed in Supplementary Table S2.

**TABLE 1:** Clinical characteristics of reports with ciprofloxacin-, levofloxacin-, and moxifloxacin-associated tendonitis and tendon rupture from the FAERS database (January 2016 to March 2021).

Characteristic	Ciprofloxacin (N, %)		Levofloxacin (N, %)		Moxifloxacin (N, %)		All fluoroquinolones	
	Tendonitis	Tendon rupture	Tendonitis	Tendon rupture	Tendonitis	Tendon rupture	Tendonitis	Tendon rupture
Number of events of tendonitis or tendon rupture	905	449	798	536	68	33	1771	1018
**Gender**								
Female	482 (53.26)	225 (50.11)	438 (54.89)	241 (44.96)	33 (48.53)	17 (51.52)	953 (53.81)	483 (47.45)
Male	380 (41.99)	203 (45.21)	248 (31.08)	240 (44.78)	30 (44.12)	15 (45.45)	658 (37.15)	458 (44.99)
Unknown	43 (4.75)	21 (4.68)	112 (14.04)	55 (10.26)	5 (7.35)	1 (3.03)	160 (9.03)	77 (7.56)
**Age (year)**								
<18	11 (1.22)	0 (0.00)	8 (1.00)	8 (1.49)	0 (0.00)	0 (0.00)	19 (1.07)	8 (0.78)
18≤ and ≤65	635 (70.16)	229 (51.00)	386 (48.37)	196 (36.57)	43 (63.24)	18 (54.54)	1064 (60.08)	443 (43.52)
>65	138 (15.25)	135 (30.07)	206 (25.81)	213 (39.74)	11 (16.18)	10 (30.30)	355 (20.05)	358 (35.17)
Unknown	121 (13.37)	85 (18.93)	198 (24.94)	119 (22.20)	14 (20.59)	5 (15.15)	333 (18.80)	209 (20.53)
Mean age (year)	49.88	58.19	57.71	63.78	52.44	60.43	53.24	61.15
**Indications**								
Infections	724 (80.00)	321 (71.49)	599 (75.06)	377 (70.34)	48 (70.59)	23 (69.70)	1371 (77.41)	721 (70.83)
Others	79 (8.73)	32 (7.13)	84 (10.53)	73 (13.62)	13 (19.12)	5 (15.15)	176 (9.94)	110 (10.81)
Product used for unknown indication	72 (7.96)	76 (16.93)	121 (15.16)	87 (16.23)	4 (5.88)	2 (6.06)	197 (11.12)	165 (16.21)
**Serious outcome**								
Death (DE)	2 (0.22)	1 (0.22)	1 (0.12)	4 (0.75)	1 (1.47)	0 (0.00)	4 (0.23)	5 (0.49)
Life-threatening (LT)	15 (1.66)	16 (3.56)	9 (1.13)	11 (2.05)	4 (5.88)	0 (0.00)	28 (1.58)	27 (2.65)
Hospitalization—initial or prolonged (HO)	150 (16.57)	126 (28.06)	100 (12.53)	149 (27.80)	9 (13.24)	14 (42.42)	259 (14.62)	289 (28.39)
Disability (DS)	461 (50.94)	186 (41.42)	360 (45.11)	219 (40.86)	20 (29.41)	10 (30.30)	841 (47.49)	415 (40.77)
Congenital anomaly (CA)	1 (0.11)	0 (0.00)	0 (0.00)	0 (0.00)	0 (0.00)	0 (0.00)	1 (0.06)	0 (0.00)
Required intervention to prevent permanent impairment/damage (RI)	4 (0.44)	5 (1.11)	13 (1.63)	11 (2.05)	0 (0.00)	0 (0.00)	17 (0.96)	16 (1.57)
Other serious (important medical event) (OT)	524 (57.90)	280 (62.36)	400 (50.12)	287 (53.54)	44 (64.70)	15 (45.45)	968 (54.66)	582 (57.17)
**Reporting region**								
Africa	0 (0.00)	0 (0.00)	3 (0.38)	2 (0.37)	0 (0.00)	0 (0.00)	3 (0.17)	2 (0.20)
Asia	2 (0.22)	2 (0.44)	12 (1.50)	10 (1.86)	4 (5.88)	1 (3.03)	18 (1.02)	13 (1.28)
Europe	497 (54.92)	193 (42.98)	314 (39.35)	162 (30.22)	21 (30.88)	10 (30.30)	832 (46.98)	365 (35.85)
North America	398 (43.98)	249 (55.46)	457 (57.27)	358 (66.79)	43 (63.24)	22 (66.67)	898 (50.71)	629 (61.79)
Oceania	2 (0.22)	0 (0.00)	1 (0.12)	1 (0.19)	0 (0.00)	0 (0.00)	3 (0.17)	1 (0.10)
South America	0 (0.00)	1 (0.22)	1 (0.12)	0 (0.00)	0 (0.00)	0 (0.00)	1 (0.06)	1 (0.10)
Unknown	6 (0.66)	0 (0.00)	10 (1.25)	3 (0.56)	0 (0.00)	0 (0.00)	16 (0.90)	3 (0.29)
**Reported person**								
Health professional								
Physician (MD)	98 (10.83)	57 (12.69)	103 (12.91)	67 (12.50)	11 (16.18)	7 (21.21)	212 (11.97)	131 (12.87)
Pharmacist (PH)	33 (3.65)	11 (2.45)	30 (3.76)	40 (7.46)	2 (2.94)	0 (0.00)	65 (3.67)	51 (5.01)
Health professional (HP)	84 (9.28)	19 (4.23)	37 (4.64)	23 (4.29)	3 (4.41)	1 (3.03)	124 (7.00)	43 (4.22)
Other health professional (OT)	129 (14.25)	77 (17.15)	98 (12.28)	62 (11.57)	13 (19.12)	9 (27.27)	240 (13.55)	148 (14.54)
Non-healthcare professional								
Consumer (CN)	516 (57.02)	252 (56.12)	459 (57.52)	298 (55.60)	33 (48.53)	13 (39.39)	1008 (56.92)	563 (55.30)
Lawyer (LW)	1 (0.11)	7 (1.56)	0 (0.00)	2 (0.37)	2 (2.94)	0 (0.00)	3 (0.17)	9 (0.88)
Unknown	44 (4.86)	26 (5.79)	71 (8.90)	44 (8.21)	4 (5.88)	3 (9.09)	119 (6.72)	73 (7.17)
Reporting year								
2021 Q1^a^	40 (4.42)	11 (2.45)	19 (2.38)	12 (2.24)	0 (0.00)	0 (0.00)	59 (3.33)	23 (2.26)
2020	178 (19.67)	49 (10.91)	123 (15.41)	79 (14.74)	11 (16.18)	4 (12.12)	312 (17.62)	132 (12.97)
2019	189 (20.88)	107 (23.83)	145 (18.17)	84 (15.67)	12 (17.64)	9 (27.27)	346 (19.54)	200 (19.65)
2018	214 (23.65)	108 (24.05)	178 (22.30)	121 (22.57)	12 (17.64)	2 (6.06)	404 (22.81)	231 (22.69)
2017	121 (13.37)	79 (17.59)	146 (18.30)	133 (24.81)	12 (17.64)	8 (24.24)	279 (15.75)	220 (21.61)
2016	163 (18.01)	95 (21.16)	187 (23.43)	107 (19.96)	21 (30.88)	10 (30.30)	371 (20.95)	212 (20.83)

a The first quarter of 2021. N, number of adverse event reported.

### Disproportionality analysis

Tendonitis and tendon rupture signals for the three fluoroquinolones based on the criteria of the four algorithms are summarized in Table 2. Ciprofloxacin had the strongest statistical association with tendonitis with the highest positive signal values (ROR 98.50, PRR 93.25, IC 6.15, and EBGM 76.80), while levofloxacin showed the strongest statistical association with tendon rupture (ROR 76.38, PRR 73.75, IC 5.84, and EBGM 63.89). Moxifloxacin was relatively weakly associated with tendonitis (ROR 28.10, PRR 27.58, IC 4.28, and EBGM 27.23) and tendon rupture (ROR 17.23, PRR 17.08, IC 3.49, and EBGM 16.95) with the lowest signal strength.

**TABLE 2:** Signal detection for ciprofloxacin-, levofloxacin-, and moxifloxacin-associated tendonitis and tendon rupture.

	N	ROR (95%CI)	PRR (χ2)	IC (IC025)	EBGM (EBGM05)
Tendonitis					
Ciprofloxacin	905	98.50 (91.52–106.02)	93.25 (67907.06)	6.15 (6.04)	76.80 (71.35)
Levofloxacin	798	92.56 (85.68–100.00)	87.80 (57748.29)	6.08 (5.97)	74.15 (68.63)
Moxifloxacin	68	28.10 (22.07–35.78)	27.58 (1719.91)	4.28 (3.93)	27.23 (21.38)
Tendon rupture					
Ciprofloxacin	449	56.49 (51.14–62.40)	55.01 (21118.00)	5.46 (5.32)	48.88 (44.25)
Levofloxacin	536	76.38 (69.64–83.78)	73.75 (33271.09)	5.84 (5.70)	63.89 (58.25)
Moxifloxacin	33	17.23 (12.22–24.32)	17.08 (495.81)	3.49 (2.98)	16.95 (12.01)

N, number of adverse event reported; PRR, proportional reporting ratio; ROR, reporting odds ratio; IC, information component; EBGM, empirical Bayes geometric mean; CI, confidence interval; 95% CI, two‐sided for ROR; χ2, chi-squared; IC025 and EBGM05, lower one‐sided for IC and EBGM, respectively.

Simultaneously, tendonitis and tendon rupture signals of ciprofloxacin, levofloxacin, and moxifloxacin in different administration routes (oral and intravenous) were detected and compared, and the results are listed in Table 3. It was observed that oral administration of ciprofloxacin, levofloxacin, and moxifloxacin showed higher frequency and stronger signal strength, suggesting a stronger statistical association with tendonitis and tendon rupture than intravenous administration.

**TABLE 3:** Relationship between oral or intravenous administration of fluoroquinolones (ciprofloxacin, levofloxacin, and moxifloxacin) and tendonitis or tendon rupture.

	N (%)	ROR (95%CI)	PRR (χ2)	IC (IC025)	EBGM (EBGM05)
Tendonitis					
Ciprofloxacin (oral)	677 (74.81)	152.35 (139.43–166.45)	146.24 (72883.92)	6.56 (6.43)	109.36 (100.09)
Ciprofloxacin (intravenous)	27 (2.98)	26.83 (18.18–39.59)	26.79 (631.06)	3.71 (3.13)	25.28 (17.13)
Levofloxacin (oral)	542(67.92)	124.44 (113.09–136.94)	120.08 (51009.26)	6.35 (6.21)	95.86 (87.12)
Levofloxacin (intravenous)	38(4.76)	42.48 (30.47–59.22)	42.37 (1408.34)	4.27 (3.78)	38.95 (27.94)
Moxifloxacin (oral)	42(61.76)	32.88 (24.20–44.68)	32.51 (1262.86)	4.18 (3.73)	32.01 (23.56)
Moxifloxacin (intravenous)	5(7.35)	22.35 (9.25–53.98)	22.32 (100.71)	2.03 (0.73)	22.09 (9.14)
Tendon rupture					
Ciprofloxacin (oral)	260(57.91)	72.26 (63.31–82.46)	71.15 (15432.12)	5.63 (5.44)	61.19 (53.61)
Ciprofloxacin (intravenous)	30(6.68)	38.09 (26.21–55.34)	38.02 (993.58)	4.01 (3.47)	35.01 (24.10)
Levofloxacin (oral)	352(65.67)	114.68 (101.98–128.96)	112.07 (31302.08)	6.17 (6.00)	90.70 (80.66)
Levofloxacin (intravenous)	46(8.58)	67.21 (49.33–91.58)	67.01 (2618.59)	43.69 (4.24)	58.79 (43.17)
Moxifloxacin (oral)	24(72.73)	27.17 (18.14–40.69)	26.99 (592.90)	3.66 (3.06)	26.65 (17.79)
Moxifloxacin (intravenous)	3(9.09)	16.66 (5.35–51.92)	16.65 (43.76)	1.34 (-0.33)	16.52 (5.30)

N, number of adverse event reported; PRR, proportional reporting ratio; ROR, reporting odds ratio; IC, information component; EBGM, empirical Bayes geometric mean; CI, confidence interval; 95% CI, two‐sided for ROR; χ2, chi-squared; IC025 and EBGM05, lower one‐sided for IC and EBGM, respectively.

Furthermore, the signal strength of fluoroquinolone-associated tendonitis and tendon rupture was calculated. After separately assessing tendonitis and tendon rupture stratified by sex and age (Supplementary Tables S3, S4), the values of all subgroups were greater than the corresponding threshold and the associations between fluoroquinolones and tendonitis and tendon rupture persisted.

### Time-to-onset of fluoroquinolone-associated tendonitis and tendon rupture

**FIGURE 2:**
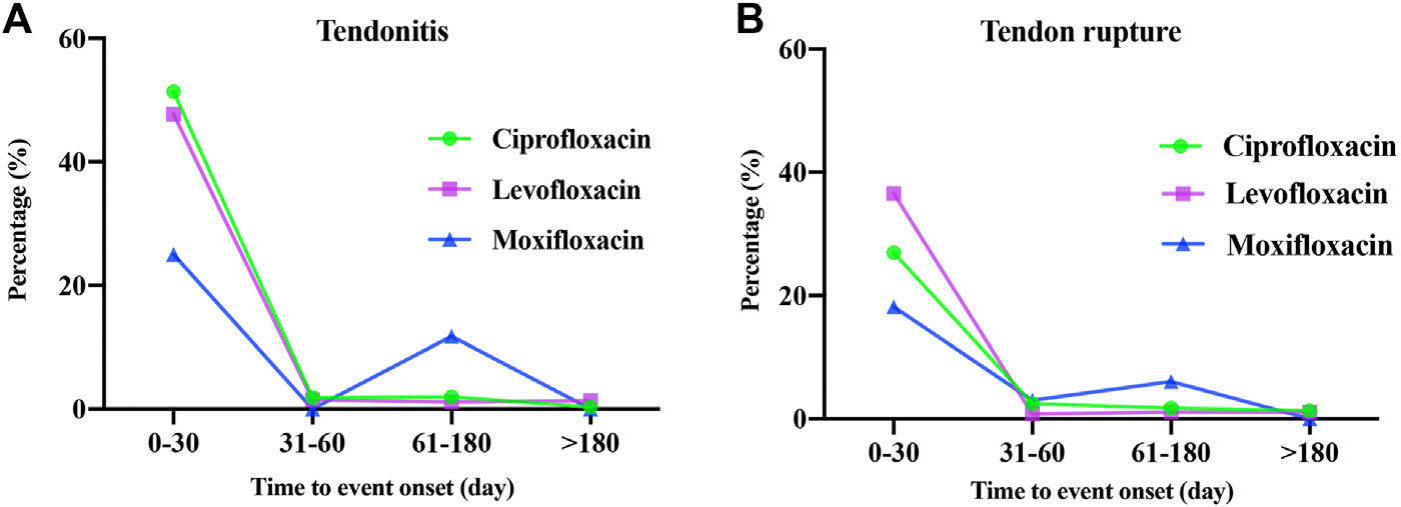
Onset time of ciprofloxacin-, levofloxacin- and moxifloxacin-associated tendonitis **(A)** and tendon rupture **(B)**.

A total of 1,311 ciprofloxacin-, levofloxacin-, and moxifloxacin-associated suspected tendonitis and tendon rupture reported the onset time. The times-to-onset of tendonitis and tendon rupture for each fluroquinolone are shown in Table 4 and Figure 2. It is noteworthy that the majority of fluroquinolone-related tendonitis and tendon rupture tended to occur within 1 month. Ciprofloxacin-related tendonitis had the shortest median onset time of 3 days [interquartile range (IQR) 1–9.5], with levofloxacin which also had 3 days as the onset time (IQR 1–7) for tendon rupture. The longest median times-to-onset were 16.5 (IQR 4–77) and 18 (12–39) days for moxifloxacin-associated tendonitis and tendon rupture, respectively.

**TABLE 4:** Onset time of ciprofloxacin-, levofloxacin-, and moxifloxacin-associated tendonitis and tendon rupture.

Interquartile range (IQR/day)	Ciprofloxacin		Levofloxacin		Moxifloxacin	
	Tendonitis	Tendon rupture	Tendonitis	Tendon rupture	Tendonitis	Tendon rupture
First quartile	1	1	1	1	4	12
Median	3	5.5	4	3	16.5	18
Third quartile	9.5	24	10	7	77	39

## Discussion

Considering the frequent use of fluroquinolones in clinical practice and the related potential risk of severe disability, this pharmacovigilance study of FAERS data highlighted the association between systematic exposure to fluoroquinolones and tendonitis and tendon rupture. Our results are consistent with previous clinical trials and literature reviews showing that fluoroquinolones may increase the risk of tendonitis and tendon rupture (Alves et al., 2019; Morales D. R. et al., 2019; Persson and Jick, 2019). A VigiBase descriptive study demonstrated that among the AEs in SOC of musculoskeletal and connective tissue disorders induced by fluoroquinolones, in addition to arthralgia (16.34%) and pain in extremity (9.98%), tendonitis (11.04%) and tendon pain (7.63%) were the most reported AEs. Levofloxacin (50.04%), ciprofloxacin (38.41%), and moxifloxacin (5.16%) were the top suspected fluoroquinolones with high reporting frequency (Huruba et al., 2021). In addition, a recent cohort study published in Taiwan, based on a nationwide population that enrolled 357,070 patients, reported that the incidence of tendon disorder increased significantly in patients with fluoroquinolone exposure compared with those without [6.61 vs. 3.34 per 10^5^ person-years, HR 1.423, 95% confidence interval (1.02,1.87), and *p* = 0.021] (Chang et al., 2022).

To the best of our knowledge, even though there have been systematic literature reviews and drug safety studies on fluoroquinolone-related tendonitis and tendon rupture before 2012, the number and content of reports are limited (van der Linden et al., 2002; van der Linden et al., 2003; Kaleagasioglu and Olcay, 2012). At present, the scientific community continues to study the risk of tendon injuries caused by fluoroquinolones mainly through pharmacoepidemiological methods, such as cohort studies (Daneman et al., 2015; Morales D. R. et al., 2019). However, our study is unique in evaluating the association of fluoroquinolones with the risk of tendonitis and tendon rupture. Based on the large-sample real-world FAERS data, we found that ciprofloxacin might be associated with the greatest risk of tendonitis, while levofloxacin associated with tendon rupture. Although moxifloxacin was reported at a lower frequency, significant AE signal strength for tendonitis and tendon rupture was still observed. The reason was that a minimum of 3,557 moxifloxacin AE reports were collected from the FAERS database, while levofloxacin and ciprofloxacin were 15,327 and 16,783, respectively. In addition, it might be because ciprofloxacin and levofloxacin were the most commonly used fluoroquinolones in clinic.

In the present study, the relationship between fluoroquinolones and suspected tendonitis and tendon rupture was slightly higher in females than in males in terms of gender proportion. The finding is consistent with a previous study that assessed the risk of quinolone-associated tendon disorders (Wise et al., 2012). However, Akali and Niranjan, (2008) found that the proportion of men is twice that of women in fluoroquinolone-related tendinopathy. Moreover, our study showed that tendonitis and tendon rupture were less likely in populations younger than 18 years than in other age groups possibly because fluoroquinolones were less commonly used in children, corresponding to previous reports (Chang et al., 2022). The average age of tendonitis and tendon rupture varied from 49.88 to 63.78 years, in accordance with the mean age reported in foreign studies (Wise et al., 2012; Alves et al., 2019). A cohort study demonstrated that patients with chronic kidney disease, diabetes, rheumatologic disease, cardiac disease, or lipid disorders and those who concomitantly used statins, aromatase inhibitors, or glucocorticoids had a significantly higher risk of tendon disorders (Chang et al., 2022). Based on the results of our FAERS data and the literature, clinicians should be particularly concerned about the use of fluoroquinolones in the elderly, especially in the presence of other comorbidities or in combination with corticosteroids.

In the present study, the majority of fluoroquinolone-associated suspected tendonitis and tendon rupture resulted in serious outcomes, such as hospitalization or prolonged hospitalization and disability. In 2019, the European Medicines Agency (EMA) and the US FDA also recommended tightening restrictions on the use of fluoroquinolones because of their disabling and potentially permanent side effects on muscles, tendons, or joints and the nervous system (US Food and Drug Administration, 2018; European Medicines Agency, 2019). Data mining on large-sample databases is valuable for generating possible AE signals in a timely manner, although they cannot provide reliable evidence of causality. However, in severe or life-threatening AE cases, regulators can revise the recommendations on the drug labels, if necessary, according to these signals under the condition of absence of deeper epidemiological or evidence-based evidence.

Fluoroquinolones are commonly administered orally or intravenously, but few studies have compared tendonitis and tendon rupture caused by the two different administrations. In our study, the vast majority of fluoroquinolones were administered orally, accounting for 94.74% (available in 1261/1331) of tendonitis and 88.95% (available in 636/715) of tendon ruptures. Tendonitis and tendon rupture caused by oral administration have stronger signal strength than those caused by intravenous administration. For example, in ciprofloxacin-induced tendonitis, the oral signal strength was ROR 152.35, PRR 146.24, IC 6.56, and EBGM 109.36, whereas the intravenous signal strength was ROR 26.83, PRR 26.79, IC 3.71, and EBGM 25.28. However, the reasons for these different results remain unclear.

Tendonitis and tendon rupture do not necessarily occur during or immediately after medication. Indeed, a significant number of AEs began within a few days or a month of fluoroquinolone initiation, and some even occurred after two or several months based on our FAERS results. In a previously published review, half of the tendon ruptures occurred within the first week of fluoroquinolone administration, and 85% of cases developed symptoms within less than a month of fluoroquinolone combined with oral corticosteroids (Akali and Niranjan, 2008). Therefore, it is suggested that clinical medical staff should focus on the AE occurrence within 1 month after the medication during the follow-up period and take appropriate treatment measures when necessary.

Fluoroquinolone-associated suspected tendon ruptures were reported by the FDA Adverse Event Reporting System in 2015 (Arabyat et al., 2015). However, our analysis is different and unique in several ways. First, the drugs in our study were the three most commonly used fluoroquinolones in clinical practice, while other fluoroquinolones were excluded (such as ofloxacin and norfloxacin). Second, our study was associated with tendonitis and tendon rupture (increased adverse events for tendonitis). Third, the risk of tendonitis and tendon rupture with different administrations, namely, oral or intravenous fluoroquinolones, was compared in our study. Fourth, we performed subgroup analysis to check the strength of the signal in male/female and different age groups. Fifth, in the present study, the AE onset time and serious outcomes of fluoroquinolone-induced tendonitis and tendon rupture were also covered, which were not found in previous studies.

Inevitably, our study has several limitations. First, FAERS data were dependent on the skills of the reporter, and missing, inadequate, excessive, selective, or incomplete reports would lead to bias. Second, it is difficult to control the interference of confounding factors such as age, dose, comorbidities, drug combinations, or other factors that may affect AEs. Third, FAERS cannot provide morbidity information because there is a lack of report on all patients using fluoroquinolones, i.e., the denominator is unknown, so it is not possible to compare the incidence of tendonitis or tendon rupture caused by different fluoroquinolones. Fourth, despite the increased number of cases of tendonitis and tendon rupture reported by fluoroquinolones, our disproportionality analysis was unable to provide a causal relationship between fluoroquinolones and the occurrence of tendonitis and tendon rupture; however, only a statistical association was provided because FAERS was not required to prove the causality between a drug and an adverse event before submitting.

## Conclusion

We explored the relationship between fluoroquinolones and tendonitis and tendon rupture from various perspectives and quantified the potential risks based on a comprehensive and systematic retrospective analysis of the FAERS database. Notably, ciprofloxacin had the greatest risk of tendonitis, while it was levofloxacin when it came to tendon rupture. Oral fluoroquinolone-induced tendonitis and tendon rupture had a stronger signal strength than intravenous administration. Furthermore, the onset time and serious outcomes of fluoroquinolone-induced tendonitis and tendon rupture were also discussed, which will be helpful for clinical practice and drug monitoring to a certain extent. Our study provided valuable references for early clinical interventions and identification of the risk of fluoroquinolone-induced tendonitis and tendon rupture.

## Data Availability

The original contributions presented in the study are included in the article/Supplementary Material; further inquiries can be directed to the corresponding author.
