# Supplementary material for: Fluoroquinolone-associated suspected tendonitis and tendon rupture: A pharmacovigilance analysis from 2016 to 2021 based on the FAERS database
Source: Front Pharmacol. 2022 Sep 6;13:990241. doi: 10.3389/fphar.2022.990241 (PMC9486157; doi:10.3389/fphar.2022.990241)
Supplement: Supplementary file 1 [file Table1.DOCX]

**Supplementary Table 1. Four major algorithms used for signal detection.**

| Algorithms | Equation | Criteria |
| --- | --- | --- |
| ROR | ROR=ad/bc | lower limit of 95% CI>1, N≥3 |
|  | 95%CI=e^ln(ROR)±1.96(1/a+1/b+1/c+1/d)^0.5^ |  |
| PRR | PRR=a(c+d)/c/(a+b) | PRR≥2, χ^2^≥4, N≥3 |
|  | χ^2^=[(ad-bc)^2](a+b+c+d)/[(a+b)(c+d)(a+c)(b+d)] |  |
| BCPNN | IC=log_2_a(a+b+c+d)/(a+c)/(a+b) | IC025>0, N≥3 |
|  | 95%CI=E(IC) ± 2V(IC)^0.5 |  |
|  | r=(a+b+c+d)^2/(a+b+1)/(a+c+1) |  |
|  | E(IC)=log_2_a(a+b+c+d)^2/(a+b+c+d+r)/(a+b)/(a+c) |  |
|  | V(IC)=1/ln2{(b+c+d+r-1)/(a+1)/(a+b+c+d+r+1)+(2+b+c+2d)/(a++b+1)/(a+b+c+d+r+3)} |  |
|  | IC025=E(IC)- 2V(IC)^0.5 |  |
| MGPS | EBGM=a(a+b+c+d)/(a+c)/(a+b) | EBGM05≥2,N≥3 |
|  | 95%CI=e^ln(EBGM)±1.96(1/a+1/b+1/c+1/d)^0.5^ |  |
|  | EBGM05=e^ln(EBGM)-1.96(1/a+1/b+1/c+1/d)^0.5^ |  |

Equation: a, number of reports containing both the suspect drug and the suspect adverse drug reaction; b, number of reports containing the suspect adverse drug reaction with other medications (except the drug of interest); c, number of reports containing the suspect drug with other adverse drug reactions (except the event of interest); d, number of reports containing other medications and other adverse drug reactions. ROR, reporting odds ratio; CI, confidence interval; N, the number of co-occurrences; PRR, proportional reporting ratio; χ^2^, chi-squared; BCPNN, Bayesian confidence propagation neural network; IC, information component; IC025, the lower limit of the 95% one-sided CI of the IC; MGPS, multi-item gamma Poisson shrinker; EBGM, empirical Bayesian geometric mean; EBGM05, the lower 95% one-sided CI of EBGM.
