# Supplementary material for: Fluoroquinolone-associated suspected tendonitis and tendon rupture: A pharmacovigilance analysis from 2016 to 2021 based on the FAERS database
Source: Front Pharmacol. 2022 Sep 6;13:990241. doi: 10.3389/fphar.2022.990241 (PMC9486157; doi:10.3389/fphar.2022.990241)
Supplement: Supplementary file 2 [file Table2.DOCX]

**Supplementary Table 2**. **Top 5 concomitant drugs for** **fluoroquinolone-associated tendonitis and tendon rupture from FAERS databases.**

| Concomitant drugs (TOP 5) | Ciprofloxacin (N) | Levofloxacin (N) | Moxifloxacin (N) |
| --- | --- | --- | --- |
| Tendonitis | Cholecalciferol (65) | Cholecalciferol (71) | Cholecalciferol (9) |
|  | Acetaminophen (56) | Albuterol (67) | Pantoprazole (8) |
|  | Vitamins (53) | Acetaminophen (65) | Levothyroxine (8) |
|  | Omeprazole (49) | Vitamins (54) | Aspirin (6) |
|  | Aspirin (38) | Aspirin (47) | Prednisone (5) |
| Tendon rupture | Acetaminophen (33) | Cholecalciferol (51) | Fluticasone (4) |
|  | Aspirin (22) | Levothyroxine (45) | Esomeprazole (4) |
|  | Cholecalciferol (21) | Aspirin (43) | Vitamins (3) |
|  | Prednisolone (21) | Atorvastatin (41) | Prednisone (3) |
|  | Vitamins (21) | Albuterol (35) | Estradiol (3) |

N, number of adverse event reports.
