# Supplementary material for: Fluoroquinolone-associated suspected tendonitis and tendon rupture: A pharmacovigilance analysis from 2016 to 2021 based on the FAERS database
Source: Front Pharmacol. 2022 Sep 6;13:990241. doi: 10.3389/fphar.2022.990241 (PMC9486157; doi:10.3389/fphar.2022.990241)
Supplement: Supplementary file 3 [file Table3.DOCX]

**Supplementary Table 3. Signal detection for fluoroquinolone-associated tendonitis and tendon rupture.**

|  | Tendonitis and Tendon rupture (N) | ROR (95% CI) | PRR (χ2) | IC (IC025) | EBGM (EBGM05) |
| --- | --- | --- | --- | --- | --- |
| ciprofloxacin | 1277 | 76.50 (71.96-81.34) | 70.76 (75498.82) | 5.86 (5.77) | 60.89 (57.28) |
| Female | 669 | 66.72 (61.35-72.56) | 61.91 (34978.10) | 5.64 (5.53) | 54.07 (49.72) |
| Male | 548 | 89.11 (81.00-98.04) | 81.22 (36165.16) | 5.91 (5.78) | 67.73 (61.56) |
| levofloxacin | 1248 | 82.05 (77.12-87.30) | 75.45 (79115.6) | 5.95 (5.86) | 65.17 (61.25) |
| Female | 625 | 86.24 (79.04-94.11) | 78.28 (42007.96) | 5.96 (5.83) | 68.99 (63.22) |
| Male | 466 | 100.29 (90.50-111.15) | 90.13 (35246.21) | 6.05 (5.91) | 77.38 (69.83) |
| moxifloxacin | 86 | 19.99 (16.12-24.78) | 19.53 (1499.59) | 3.98 (3.67) | 19.36 (15.61) |
| Female | 41 | 17.69 (12.96-24.14) | 17.30 (625.56) | 3.60 (3.14) | 17.17 (12.58) |
| Male | 39 | 23.64 (17.15-32.57) | 22.96 (32.57) | 3.84 (3.37) | 22.69 (16.47) |

N, number of adverse event reports; PRR, the proportional reporting ratio; ROR, the reporting odds ratio; IC, the information component; EBGM, the empirical Bayes geometric mean; CI, confidence interval; 95% CI, two‐sided for ROR, χ2, chi-squared; IC025 and EBGM05 lower one‐sided for IC and EBGM.
