# Supplementary material for: Fluoroquinolone-associated suspected tendonitis and tendon rupture: A pharmacovigilance analysis from 2016 to 2021 based on the FAERS database
Source: Front Pharmacol. 2022 Sep 6;13:990241. doi: 10.3389/fphar.2022.990241 (PMC9486157; doi:10.3389/fphar.2022.990241)
Supplement: Supplementary file 4 [file Table4.DOCX]

**Supplementary Table 4. Signal detection for fluoroquinolone-associated tendonitis and tendon rupture.**

|  | Tendonitis and Tendon rupture (N) | ROR (95% CI) | PRR (χ2) | IC (IC025) | EBGM (EBGM05) |
| --- | --- | --- | --- | --- | --- |
| ciprofloxacin | 1277 | 76.50 (71.96-81.34) | 70.76 (75498.82) | 5.86 (5.77) | 60.89 (57.28) |
| 18≤and≤65 (years) | 819 | 77.69 (71.76-84.12) | 69.40 (45195.78) | 5.73 (5.62) | 56.88 (52.54) |
| >65 (years) | 256 | 52.45 (45.84-60.01) | 49.28 (10607.5) | 5.21 (5.02) | 43.23 (37.79) |
| levofloxacin | 1248 | 82.05 (77.12-87.30) | 75.45 (79115.6) | 5.95 (5.86) | 65.17 (61.25) |
| 18≤and≤65 (years) | 554 | 73.11 (66.58-80.28) | 65.25 (30771.71) | 5.70 (5.56) | 57.30 (52.18) |
| >65 (years) | 383 | 99.01 (88.15-111.21) | 88.89 (27087.18) | 5.93 (5.77) | 72.43 (64.48) |
| moxifloxacin | 86 | 19.99 (16.12-24.78) | 19.53 (1499.59) | 3.98 (3.67) | 19.36 (15.61) |
| 18≤and≤65 (years) | 52 | 19.43 (14.71-25.66) | 18.78 (866.68) | 3.77 (3.36) | 18.57 (14.06) |
| >65 (years) | 17 | 13.96 (8.62-22.61) | 13.71 (198.98) | 2.92 (2.23) | 13.61 (8.40) |

N, number of adverse event reports; PRR, the proportional reporting ratio; ROR, the reporting odds ratio; IC, the information component; EBGM, the empirical Bayes geometric mean; CI, confidence interval; 95% CI, two‐sided for ROR, χ2, chi-squared; IC025 and EBGM05 lower one‐sided for IC and EBGM.
